# A longitudinal multi-omic dataset of pediatric cystic fibrosis patients receiving lumacaftor/ivacaftor therapy: clinical, microbiome, inflammatory and metabolomic measurements collected over 24 months

**DOI:** 10.1016/j.dib.2026.113237

**Published:** 2026-09-10

**Authors:** Rebecca Luise Knoll, Virginia Rossow, Juliane Rössler, Katja Hilbert, Víctor Hugo Jarquín-Díaz, Theda Ulrike Patricia Bartolomaeus, Oliver Nitsche, Morgan Essex, Ulrike Löber, Chen Meng, Karin Kleigrewe, Stephan Gehring, Sofia Kirke Forslund-Startceva, Krystyna Poplawska

**Affiliations:** a Children’s Hospital, University Medical Center of the Johannes Gutenberg University Mainz, Langenbeckstraße 1, 55131, Mainz, Germany; b Charité – Universitätsmedizin Berlin, Freie Universität Berlin and Humboldt-Universität zu Berlin, Charitéplatz 1, 10117, Berlin, Germany; c Max Delbrück Center for Molecular Medicine in the Helmholtz Association (MDC), Robert-Rössle-Straße 10, 13125, Berlin, Germany; d Experimental and Clinical Research Center, Max Delbrück Center for Molecular Medicine and Charité – Universitätsmedizin Berlin, Lindenberger Weg 80, 13125, Berlin, Germany; e Division of Gastroenterology and Benioff Center for Microbiome Medicine, Department of Medicine, University of California San Francisco, 513 Parnassus Avenue, San Francisco, CA, 94143, USA; f German Centre for Cardiovascular Research (DZHK), Partner Site Berlin, Charitéplatz 1, 10117, Berlin, Germany; g Bavarian Center for Biomolecular Mass Spectrometry, TUM School of Life Sciences, Technical University of Munich (TUM), Gregor-Mendel-Straße 4, 85354, Freising, Germany; h German Center for Cardiovascular Research (DZHK), partner site Berlin, Berlin, Germany; i Klinik für Pädiatrische Pneumologie und Allergologie, Waldburg-Zeil Kliniken, Fachkliniken Wangen, Am Vogelherd 14, 88239, Wangen im Allgäu, Germany; j Cluster of Excellence ImmunoPreCept (EXC 3118), Charité-Universitätsmedizin Berlin, Berlin, Germany

**Keywords:** Pediatric cystic fibrosis, Lumacaftor/ivacaftor, Deep phenotyping, Microbiome, Metabolome

## Abstract

This article describes a longitudinal multiomic dataset generated within a prospective phase IV pilot study of eight children with cystic fibrosis homozygous for the F508del mutation who initiated lumacaftor/ivacaftor therapy. Participants were followed for up to 24 months with repeated collection of clinical metadata, anthropometric measurements, sweat chloride concentrations, lung function assessments, inflammatory markers, conventional microbiology results, stool samples, respiratory samples, and serum metabolomics. The resulting dataset links host phenotypes, microbiome composition, inflammatory parameters, and metabolomic measurements across multiple body sites and time points. Microbiome data were generated from stool, sputum and throat swab samples using 16S rRNA gene sequencing, while serum metabolomics was assessed using untargeted mass spectrometry. The dataset is publicly available through SRA, MassIVE and GitHub repositories and may support future studies of longitudinal host–microbiome interactions, biomarker discovery, methodological benchmarking and comparative analyses across CFTR modulator eras.

Specifications Table

**Table:** 

Subject	Biology
Specific subject area	Microbiology: microbiome; Medicine: Cystic fibrosis Multi-omic (Microbiome, Metabolome, inflammatory markers, clinical metadata) 24‑month longitudinal dataset from children with cystic fibrosis undergoing CFTR-modulator therapy (Lumacaftor/Ivacaftor)
Type of data	Table, Figure Raw, Analyzed, Filtered, Processed
Data collection	Spirometry, sweat testing, routine clinical laboratory analyses, 16S rRNA gene sequencing, conventional microbiology, untargeted mass spectrometry; performed on samples/subjects every 3 months for a maximum duration of 24 months; right before and during Lumacaftor/Ivacaftor therapy.
Data source location	City/Town/Region: Mainz Country: Germany Latitude and longitude: **49.99° N latitude** and **8.25° E longitude**
Data accessibility	**Repository 1** Repository name: GitHub – DeepPheno_Luma-Iva Data identification number (or DOI or persistent identifier): 10.5281/zenodo.20,837,397 Direct URL to data: https://github.com/RebeccaLuise/DeepPheno_Luma-Iva Instructions for accessing these data: Source code, analysis scripts, and documentation are publicly available and can be accessed directly through the GitHub repository without restrictions. **Repository 2** Repository name: NCBI Sequence Read Archive (SRA) Data identification number (or DOI or persistent identifier): BioProject accession PRJNA1119982 Direct URL to data: https://www.ncbi.nlm.nih.gov/bioproject/PRJNA1119982 Instructions for accessing these data: Raw sequencing data are publicly available through the NCBI Sequence Read Archive under BioProject accession PRJNA1119982 and can be downloaded directly from the repository without restrictions. **Repository 3** Repository name: MassIVE Data identification number (or DOI or persistent identifier): MSV000094922 Direct URL to data: https://massive.ucsd.edu/ProteoSAFe/dataset.jsp?task=2316013213f844eca77ead32ef69f9a7
	Instructions for accessing these data: Proteomics mass spectrometry data are publicly available through the MassIVE repository under accession MSV000094922 and can be downloaded directly without restrictions.
Related research article	none

## 1 Value of the Data

• Provides a rare 24‑month longitudinal pediatric cystic fibrosis dataset.
• Integrates gastrointestinal, respiratory, and systemic measurements within the same participants with paired stool, throat swab, sputum, blood, sweat chloride, and clinical metadata.
• Includes three microbiome habitats (stool, sputum and throat swabs)
• Provides an opportunity to compare pre-ETI and early modulator-era pediatric cohorts and/or enables reuse for microbiome, metabolomics, biomarker, and systems medicine studies.

## 2 Background

The original study was designed as a pilot phase IV observational cohort to establish infrastructure and sampling workflows for future large-scale pediatric cystic fibrosis studies documenting the impact of CFTR-modulator therapy on participants health and microbiomes. The resulting dataset combines repeated clinical assessments with microbiome, inflammatory and metabolomic measurements. Because of the breadth of phenotyping and longitudinal sampling, the dataset has value beyond the original study objectives and is therefore being made available as a reusable resource.

## 3 Data Description

The dataset is distributed across three repositories. Processed clinical, microbiome, metabolomics, and metadata files together with analysis scripts are available in the GitHub repository (DeepPheno_Luma-Iva). Raw 16S rRNA gene sequencing data are deposited in the NCBI Sequence Read Archive (PRJNA1119982), and raw metabolomics mass spectrometry data are available through MassIVE (MSV000094922). The GitHub repository contains processed metadata tables, microbiome sequencing objects, metabolomics feature tables, and scripts used for data processing and figure generation.

The dataset consists of longitudinal measurements from eight children with cystic fibrosis homozygous for the F508del mutation who were followed for up to 24 months. Fig. 1 provides an overview of the study design and sampling schedule. Clinical metadata, anthropometric measurements, sweat chloride concentrations, blood samples, stool samples, sputum samples, and throat swabs were collected at repeated visits. While all participants completed the first 12 months of follow-up, sample availability decreased during later visits due to COVID-19 restrictions and participant availability. Table 1 summarizes cohort characteristics and visit completeness.

**Fig. 1:**
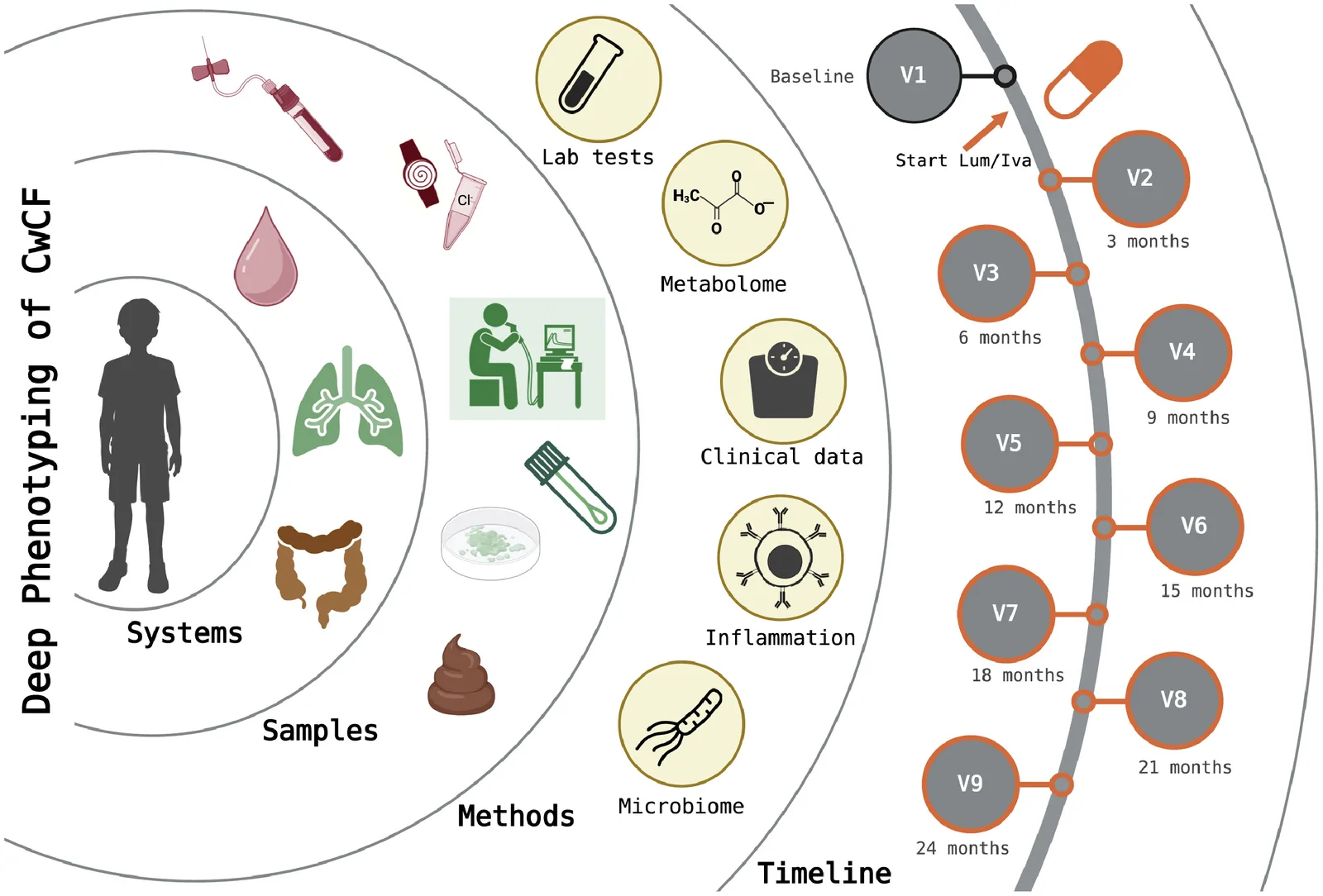
Study design and sampling framework. Overview of the longitudinal study design and sample collection schedule. Clinical metadata and biospecimens (stool, sputum, throat swabs, blood, and sweat) were collected during up to nine study visits over a 24-month period. Measurements included lung function testing, routine clinical laboratory parameters, inflammatory markers, microbiome profiling by 16S rRNA gene sequencing, and untargeted serum metabolomics. Figure created with BioRender®.

**Table 1:** Characteristics at baseline and across visits.

Visit	1	2	3	4	5	6	7	8	9
Months (mos.) from tt start	Baseline	3 mos.	6 mos.	9 mos.	12 mos.	15 mos.	18 mos.	21 mos.	24 mos.
N participants	N = 8	N = 8	N = 8	N = 8	N = 8	N = 5	N = 7	N = 4	N = 5
Sex									
female^1^	5 (63%)	5 (63%)	5 (63%)	5 (63%)	5 (63%)	3 (60%)	5 (71%)	4 (100%)	4 (80%)
male^1^	3 (38%)	3 (38%)	3 (38%)	3 (38%)	3 (38%)	2 (40%)	2 (29%)	0 (0%)	1 (20%)
Age (years)	9.50 (7.00, 10.25)	9.50 (7.00, 10.25)	9.50 (7.00, 10.25)	9.50 (7.00, 10.25)	10.00 (8.00, 10.50)	10.00 (10.00, 11.00)	10.00 (7.00, 10.50)	7.00 (6.50, 7.75)	10.00 (7.00, 10.00)
BMI z-score	−0.68 (−1.10, −0.19)	−0.61 (−0.73, −0.02)	−0.71 (−0.87, −0.40)	−0.48 (−0.74, −0.26)	−0.47 (−0.72, −0.20)	−0.45 (−0.50, −0.45)	−0.11 (−0.51, −0.07)	−0.18 (−0.73, 0.19)	−0.36 (−0.47, −0.11)
Lung function									
ppFEV1	92 (85, 107)	100 (92, 103)	96 (89, 104)	93 (89, 103)	97 (92, 109)	87 (81, 97)	92 (88, 95)	95 (89, 112)	86 (85, 96)
ppFVC	100 (93, 103)	105 (98, 108)	98 (91, 110)	97 (92, 108)	100 (95, 112)	98 (88, 108)	96 (92, 106)	103 (94, 117)	95 (94, 106)
Sweat chloride [mmol/l]	101 (94, 104)	75 (70, 80)			74 (69, 78)				
Conventional microbiology results									
Throat: Staphylococcus aureus*	5/8 (63%)	7/8 (88%)	6/8 (75%)	5/8 (63%)	4/6 (67%)	3/5 (60%)	4/6 (67%)	2/4 (50%)	3/5 (60%)
Sputum: Staphylococcus aureus*	3/5 (60%)	2/6 (33%)	2/6 (33%)	3/4 (75%)	3/4 (75%)	3/4 (75%)	3/5 (60%)		2/3 (67%)

1 n (%); Median (IQR).

⁎ positive /N samples, tt = treatment, ppFEV1= percent predicted forced expiratory volume in 1 s, ppFVC = percent predicted functional vital capacity.

Systemic measurements are shown in Fig. 2. Sweat chloride concentrations were available at baseline, 3 months, and 12 months. Longitudinal measurements of serum IgG and leukocyte counts are shown for individual participants. Untargeted metabolomics data were generated from serum samples and are represented by longitudinal abundance profiles of selected metabolites, including ivacaftor and tryptophan.

**Fig. 2:**
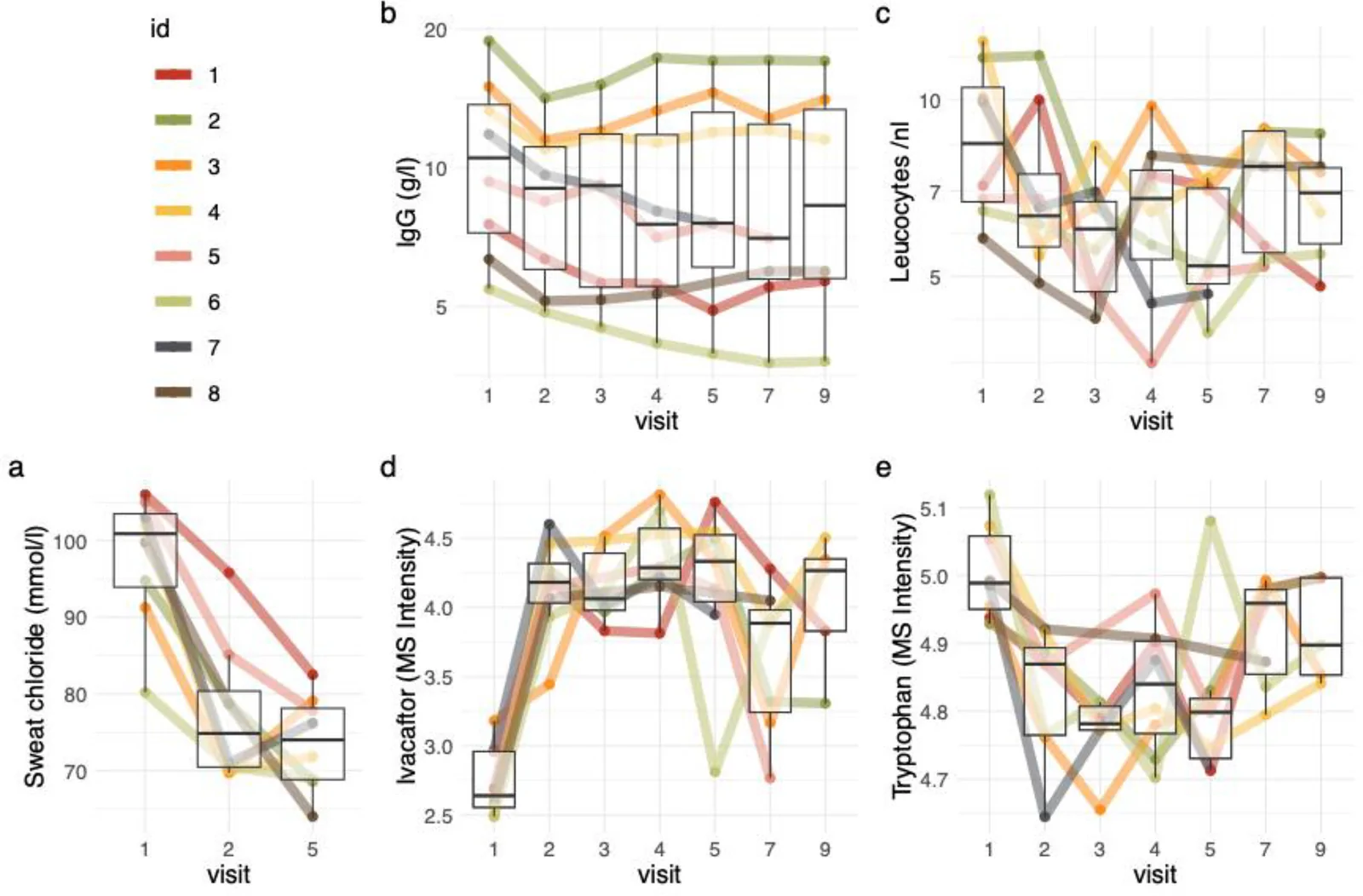
Longitudinal systemic measurements. Systemic measurements collected during follow-up. (a) Sweat chloride concentrations measured at baseline, month 3, and month 12. (b) Serum IgG concentrations. (c) Blood leukocyte counts. (d–e) Log10-transformed metabolite intensities obtained by untargeted serum metabolomics. Blood-derived measurements shown in panels (b–e) are not available for visits 6 and 8 because blood samples were not collected at these visits. Colors indicate individual participants and are consistent across all figures. Statistical analyses were performed using exploratory linear mixed-effects models with participant identifier included as a random effect.

Gastrointestinal measurements are summarized in Fig. 3. BMI z-scores, fecal calprotectin concentrations, and liver enzyme measurements were collected repeatedly throughout follow-up. Stool microbiome profiles generated by 16S rRNA gene sequencing are represented by alpha-diversity measurements and Bray–Curtis-based ordination analyses.

**Fig. 3:**
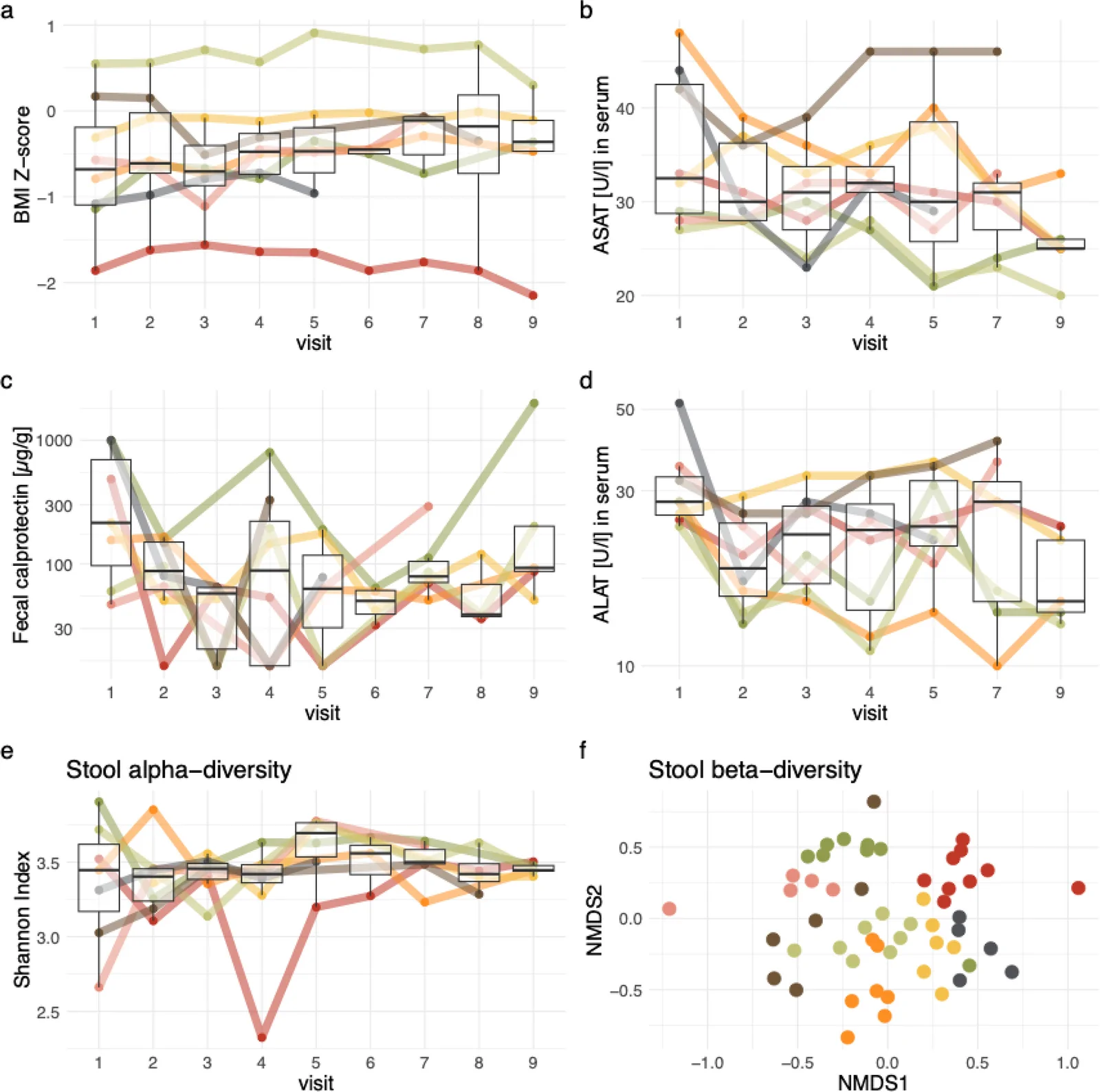
Longitudinal gastrointestinal measurements and stool microbiome profiles. Gastrointestinal measurements obtained during follow-up. (a) BMI z-scores. (b) Fecal calprotectin concentrations. (c) Aspartate aminotransferase (ASAT) concentrations. (d) Alanine aminotransferase (ALAT) concentrations. Blood-derived measurements shown in panels (b–d) are not available for visits 6 and 8 because blood samples were not collected at these visits. (e) Stool microbiome alpha diversity calculated as Shannon diversity index. (f) Stool microbiome beta diversity visualized using non-metric multidimensional scaling (NMDS) of Bray–Curtis dissimilarities derived from 16S rRNA gene sequencing data. Colors indicate individual participants.

Respiratory measurements are shown in Fig. 4. Lung function data, expressed as changes in ppFEV1 relative to baseline, were collected longitudinally. Sputum inflammatory markers were available for a subset of participants able to provide sputum samples. Respiratory microbiome data were obtained from both sputum and throat swab samples and are represented by taxonomic composition and ordination analyses.

**Fig. 4:**
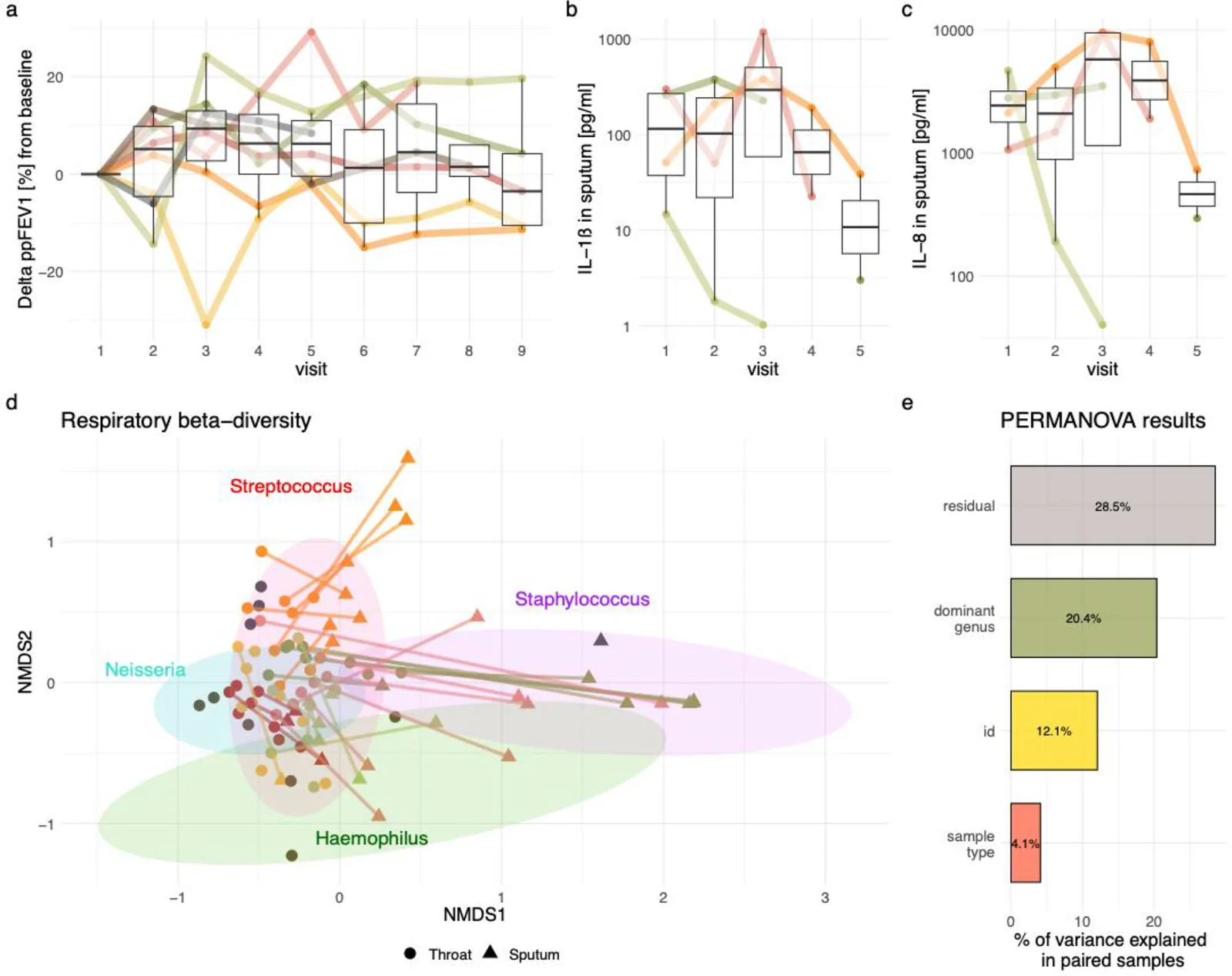
Longitudinal respiratory measurements and respiratory microbiome profiles. Respiratory measurements and microbiome data collected during follow-up. (a) Relative change in ppFEV1 compared with baseline measurements. (b–c) Sputum inflammatory marker concentrations. (d) Principal coordinate analysis of respiratory microbiome composition based on Bray–Curtis dissimilarities calculated from sputum and throat swab 16S rRNA gene sequencing data. Colors indicate participants and shapes indicate sample type. Paired samples from the same visit are connected by lines. (e) Variance explained (R²) by selected variables in PERMANOVA models of paired respiratory microbiome samples.

In addition to the measurements shown in the main figures, the dataset includes conventional microbiology results, complete blood counts, inflammatory markers, medication records, antibiotic exposure data, and technical metadata related to microbiome sequencing and metabolomics processing. Together, the repositories provide access to raw sequencing data, raw metabolomics data, processed analytical datasets, metadata tables, and reproducible analysis workflows.

## 4 Experimental Design, Materials and Methods

### 4.1 Participants and ethics

All participants were recruited from the Pediatric Pulmonology Outpatient Clinic at the University Medical Center Mainz. Study participants and their parents provided informed written consent prior to study inclusion. The study design was approved by the local ethical committee (Landesärztekammer Rheinland-Pfalz 2018–13,526) and participants were recruited in accordance with the principles of the Declaration of Helsinki. The study was registered in the German Clinical Trials Register (DRKS00022267).

### 4.2 Sampling and sample processing

Sputum was induced using physiotherapy and 6% saline inhalation at each visit, though not all study participants could expectorate. Sputum was processed within two hours, separating saliva by ensuring squamous epithelial cells were <10%. Sputum was diluted with DTT (0.1%) and PBS, filtered (75 µm), centrifuged at 750 G for 10 min, and stored at −80 °C, with some supplemented with protease inhibitors. Processing followed Eckrich et al., 2017 [1].

Deep throat swabs were collected by nurses, frozen immediately at −80 °C. Stool samples were collected at home, refrigerated for up to 24 h, and frozen at −80 °C, following protocol [2]. Blood samples were taken during routine check-ups (Visit 1–5, 7, 9) and sent for clinical analysis, with remaining serum frozen at −80 °C. Sweat chloride was measured at V1, V2, and V5 using Pilocarpine Iontophoresis.

### 4.3 Lung function

The determination of FEV1 (Forced Expiratory Volume in one second) and FVC (Forced Vital Capacity) was conducted using plethysmography with the MasterScreenTM Body equipment from Vyaire. Standard procedures outlined in the European Respiratory Society Technical Statement [3], were followed.

### 4.4 Conventional microbiology

Conventional microbiology on sputum and throat samples was performed by the Institute of Medical Microbiology and Hygiene Mainz following established protocols for CF respiratory specimens. Sputum was liquefied using 0.1% DTT, then inoculated onto various agar media via the 3-loop streak method. Culture conditions varied based on incubation time and temperature, such as chocolate agar plates incubated with 5% CO2 for Neisseria and Sabouraud plates at 30 °C for mold detection. The media used are given in the supplementary material section.

### 4.5 Microbiome analysis

Sputum samples were treated with Benzonase to remove extracellular human DNA. DNA from sputum, throat swabs, and stool was extracted using DNeasy PowerSoil Pro Kits (QIAGEN, Germany). Cell lysis was performed by bead-beating (30 Hz, 2 cycles of 7 min) on a TissueLyser.

The V4 region of the 16S rRNA gene was amplified using 515f and 806r primers, with indexes added using the Nextera XT DNA Library Preparation Kit (Illumina, USA). Sequencing was conducted on a MiSeq platform (Illumina, USA) using V3 chemistry. Technical metadata (e.g., extraction date, DNA quantity) was recorded, and negative controls were sequenced for quality control. Amplicon Sequence Variants (ASVs) were identified, and taxonomy was assigned using DADA2 [4] and the SILVA database [5]. Rarefaction analyses ensured adequate sequencing depth, and samples with <2000 reads were excluded.

### 4.6 Inflammation profiling

Sputum inflammation was evaluated using a cytometric bead array (CBA) and neutrophil elastase (NE) measurements. The CBA (BD™ CBA Human Inflammatory Cytokines Fix Set) measured TNFα, IL-1β, IL-6 and IL-8 concentrations in sputum supernatant via a Fluorescence-Activated Cell Sorter (FACS) (BD LSR II; BD Biosciences) with data processed using FCAP Array Software v3.0 (BD Biosciences).

Neutrophile elastase (NE) concentration was determined using a chromogenic substrate assay at 405 nm. As a negative control, sputum was mixed with the competitive human NE inhibitor Sivelestat (Merck KGaA, Darmstadt, Germany). The analysis was performed using a Fluorescence Plate Reader (Tecan Spark; Tecan Trading AG, Switzerland). The analysis method was adapted from Dittrich et al. [6].

Systemic inflammation markers (CRP, immunoglobulins, liver enzymes) and blood parameters (leukocyte count, ESR) were analyzed at the University Medical Center Mainz. Gut inflammation was assessed by measuring fecal calprotectin using the Quantum Blue® assay.

### 4.7 Metabolomics

We employed untargeted mass spectrometry (MS) to profile the serum metabolome at each timepoint [7]. Samples were analyzed using hydrophilic interaction chromatography (HILIC) for polar metabolites and reversed phase (RP) chromatography for mid-polar metabolites in the negative and positive ionization mode (HILIC negative, HILIC positive, RP negative, and RP positive). Each generated data set (HILIC negative, HILIC positive, RP negative, and RP positive) was analyzed separately. To ensure robustness, only metabolites annotated based on MS1 and MS2 spectral match to in-house and external databases were included in the statistical analysis. Differences in metabolome profiles resulting from treatment were evaluated using both multivariate and univariate analyses, after removal of the quality control samples. The mass spectrometric data is available on https://massive.ucsd.edu under the accession number MassIVE MSV000094922.

## Limitations

The dataset originates from a pilot cohort and should not be interpreted as representative of the broader cystic fibrosis population. Follow-up completeness decreased after month 12, and sputum availability varied because younger participants could not consistently expectorate samples. Despite these limitations, the cohort provides unusually dense longitudinal phenotyping across multiple biological domains and remains valuable as a reusable data resource.

## Ethics Statement

The study design was approved by the local ethical committee (Landesärztekammer Rheinland-Pfalz 2018–13,526) and participants were recruited in accordance with the principles of the Declaration of Helsinki. The study was registered in the German Clinical Trials Register (DRKS00022267).

## Declaration of Generative AI and AI-Assisted Technologies in the Manuscript Preparation Process

During the preparation of this work, the author(s) used ChatGPT 5.5 for language editing and reformatting to the journal’s requirements. The author(s) reviewed and edited the output as needed and take full responsibility for the content of the published article.

## CRediT authorship contribution statement

**Rebecca Luise Knoll:** Conceptualization, Methodology, Investigation, Data curation, Formal analysis, Visualization, Writing – original draft, Writing – review & editing. **Virginia Rossow:** Formal analysis, Data curation, Writing – review & editing. **Juliane Rössler:** Investigation, Data curation. **Katja Hilbert:** Investigation, Data curation. **Víctor Hugo Jarquín-Díaz:** Formal analysis, Data curation, Writing – review & editing. **Theda Ulrike Patricia Bartolomaeus:** Formal analysis, Data curation, Writing – review & editing. **Oliver Nitsche:** Investigation, Data curation. **Morgan Essex:** Formal analysis, Data curation, Writing – review & editing. **Ulrike Löber:** Formal analysis, Data curation, Writing – review & editing. **Chen Meng:** Investigation, Formal analysis, Data curation, Writing – review & editing. **Karin Kleigrewe:** Investigation, Formal analysis, Data curation, Writing – review & editing. **Stephan Gehring:** Conceptualization, Supervision, Writing – review & editing. **Sofia Kirke Forslund-Startceva:** Conceptualization, Formal analysis, Data curation, Supervision, Writing – review & editing. **Krystyna Poplawska:** Conceptualization, Formal analysis, Data curation, Writing – original draft, Writing – review & editing, Supervision.

## Data Availability

GitHubDeepPheno_Luma-Iva (Original data). MassIVEOrkambiKIDS: Impact of CFTR-modulation with Lumacftor/Ivacaftor on the intestinal and respiratory microbiome and microbial metabolites in children aged 2 to 12 with cystic fibrosis (Original data). NCBI Sequence Reads ArchiveOrkambiKIDS: Impact of CFTR-modulation with Lumacftor/Ivacaftor on the intestinal and respiratory microbiome and microbial metabolites in children aged 2 to 12 with cystic fibrosis (Original data).
